# Increased cerebral functional connectivity in ALS: A resting-state magnetoencephalography study

**DOI:** 10.1212/WNL.0000000000005333

**Published:** 2018-04-17

**Authors:** Malcolm Proudfoot, Giles L. Colclough, Andrew Quinn, Joanne Wuu, Kevin Talbot, Michael Benatar, Anna C. Nobre, Mark W. Woolrich, Martin R. Turner

**Affiliations:** From the Nuffield Department of Clinical Neurosciences (M.P., K.T., M.R.T.), and Oxford Centre for Human Brain Activity (M.P., G.L.C., A.Q., A.C.N., M.W.W., M.R.T.), University of Oxford, UK; and Miller School of Medicine (J.W., M.B.), University of Miami, FL.

## Abstract

**Objective:**

We sought to assess cortical function in amyotrophic lateral sclerosis (ALS) using noninvasive neural signal recording.

**Methods:**

Resting-state magnetoencephalography was used to measure power fluctuations in neuronal oscillations from distributed cortical parcels in 24 patients with ALS and 24 healthy controls. A further 9 patients with primary lateral sclerosis and a group of 15 asymptomatic carriers of genetic mutations associated with ALS were also studied.

**Results:**

Increased functional connectivity, particularly from the posterior cingulate cortex, was demonstrated in both patient groups compared to healthy controls. Directionally similar patterns were also evident in the asymptomatic genetic mutation carrier group.

**Conclusion:**

Increased cortical functional connectivity elevation is a quantitative marker that reflects ALS pathology across its clinical spectrum, and may develop during the presymptomatic period. The amelioration of pathologic magnetoencephalography signals might be a marker sensitive enough to provide proof-of-principle in the development of future neuroprotective therapeutics.

Amyotrophic lateral sclerosis (ALS) is an adult-onset neurodegenerative disease of the motor system. Neuroprotective strategies, particularly considering presymptomatic ALS, will require proof-of-principle biomarkers.^1^

Resting-state networks (RSNs) reflect the functional organization of the brain^2^; RSN disturbances are associated with diverse neurodegenerative diseases,^3,4^ altering functional connectivity (FC) (the statistical dependency in activity between remote sites^5^). The correspondence between RSN topography in health, and spatial patterns of both pathologic atrophy and protein deposition, suggests RSNs reflect the neuroanatomical substrate of diseases.^6^

MRI resting-state activity (rs-fMRI) exposes significant increases in FC among patients with ALS, correlating with progression.^7^ While this may be compensatory, cortical hyperexcitability is a characteristic feature of ALS,^8^ perhaps reflecting loss of inhibitory interneuronal influence,^9^ in turn associated with increased FC.^10^ A specific spatial pattern of increased FC was demonstrated in asymptomatic gene carriers at high risk of ALS,^11^ suggesting increased FC as an early pathologic feature amenable to neuroprotective strategies.

Recent EEG studies have also described FC increases,^12^ alongside global reorganization of inferred cortical network topology.^13^ Magnetoencephalography (MEG) extends the localization capabilities of noninvasive neurophysiology,^14^ negating dispersive effects of skull and scalp. The temporal resolution of MEG offers advantages, relevant in the assessment of dynamic cognitive processes, such as movement preparation, clinically affected by neurodegeneration.^15^ MEG simultaneously can explore degenerating cortical networks^16^; MEG-derived RSNs appear consistent with rs-fMRI.^17^ We therefore applied resting-state MEG to investigate increased FC as a consistent feature of ALS pathogenesis, to consider the clinical implications of FC topography in ALS and to explore FC structure before symptoms manifest.

## Methods

### Participants

All patients were recruited from the Oxford Motor Neurone Disease Care and Research Centre, diagnosed by experienced neurologists according to standard criteria (minimum El Escorial probable at enrollment, and subsequently followed longitudinally to confirm expected disease progression). A range of patients with ALS, plus a small group with the consistently slowly progressive upper motor neuron–only variant, primary lateral sclerosis (PLS), were included after the necessary exclusion of any credible mimic disorder. Asymptomatic carriers of ALS-linked genetic mutations (AGCs) (*C9orf72* and *SOD1*) were recruited through contact with the Oxford Centre or through prior enrollment in the Pre-fALS Study based at the University of Miami.^18^ Healthy controls were distributed into age-matched groups.

Physical and brief cognitive assessments were undertaken on the day of MEG, the latter using initially the revised Addenbrooke’s Cognitive Examination, later superseded by the Edinburgh Cognitive and Behavioural ALS Screen. Details of participants included in the final analysis are given in the table.

**Table:**
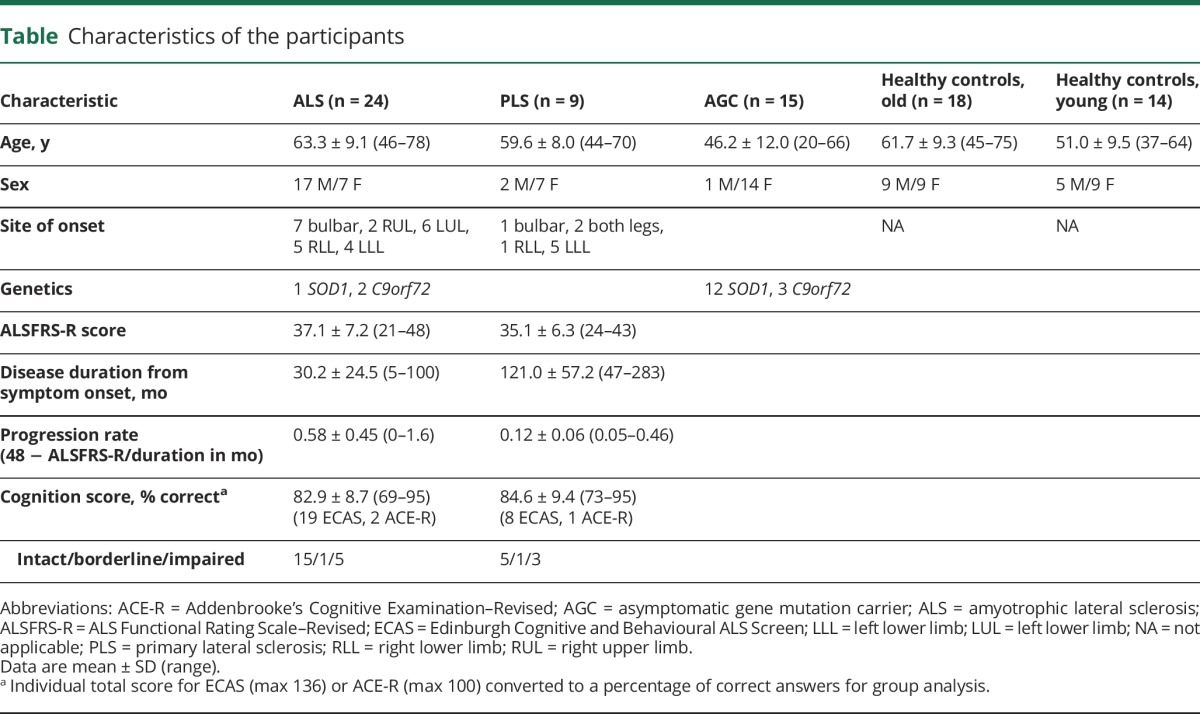
Characteristics of the participants

### MEG acquisition

Ten continuous minutes of MEG resting-state data were acquired per participant on an Elekta Neuromag 306-channel scanner (Elekta AB, Stockholm, Sweden) at the Oxford Centre for Human Brain Activity. The participant was seated comfortably in the scanner and instructed to remain still and awake, but without performing any specific task. Immediately prior to acquisition, a Polhemus 3D tracking system (Polhemus, Colchester, Vermont) recorded 4 fixed coil positions relative to nasion and preauricular fiducial landmarks, alongside distributed points (∼100) covering the scalp and in later cases the nose (∼50 points) surface. Coils were intermittently energized to obtain a continuous record of head position with the MEG helmet. The recording was made in the “eyes open” state with participants' visual fixation directed to a black cross printed on white paper 90 cm away. Blinks and saccades were monitored continuously using a combination of surface electrooculography and infrared eye tracker. ECG was monitored at the wrists. Participants underwent a structural MRI for coregistration purposes, typically on the same day as MEG (or within 1 month), using a Siemens Trio 3T (Siemens, Munich, Germany) (3-dimensional, whole-brain, T1-weighted using a magnetization-prepared rapid-acquisition gradient echo sequence; repetition time/echo time = 2,040 milliseconds/4.7 milliseconds; flip angle = 8°; 1-mm isotropic resolution; 6-minute acquisition time).

### Preprocessing

Raw MEG data were converted to SPM8 format and inspected to identify bad channels before passing the raw MEG data through MaxFilter v2.2 (-ST extension, thus creating a set of virtual channels after both signal space separation and continuous head movement compensation). Post MaxFilter, the filtered data were converted to SPM8, down-sampled to 250 Hz, and again visually inspected. Bad segments were manually marked in the continuous data stream. Data were then cleaned using a semiautomated SPM8 script as follows. Data were initially high-pass filtered at 0.3 Hz. The independent component analysis (ICA) components corresponding to physiologic artifact generated by blink and cardiac cycle were identified and removed from the data. Coregistration was then performed using either SPM8 or a bespoke version of the SPM8 coregistration tool adapted to include the registration of headshape points on the nose (collected in the latter portion of the study when this algorithm was released). Coregistration fit was checked for each individual, and errant headshape points were manually removed if necessary before repetition. Data were then source-transformed using a linearly constrained minimum variance beamformer, which combines information over both magnetometers and gradiometers and uses principal component analysis to regularize the data covariance matrix estimation and account for the reduction in dimensionality caused by MaxFilter, with a single-shell forward model into MNI (Montreal Neurological Institute) space at a 3-dimensional gridstep of 6 mm.^19^ Source-space covariance maps were created in NIfTII format and inspected for each individual using the FSL tool *FSLview*. At this stage, a limited number of participants were identified as having suboptimal source-space MEG data, either because of remaining contamination by artifact or failure of the beamformer algorithm to perform adequately. Raw data were reinspected for these individuals but no correctable source of noise was typically identified; it was noted that several individuals without structural MRI available for coregistration fell into this category. Participants unable to complete MRI examinations (because of claustrophobia, respiratory insufficiency, or other contraindication) were therefore excluded. In total, the number of additional participants excluded from further analysis because of qualitatively suboptimal source-space datasets were 1 ALS, 1 PLS, 2 AGCs, and 1 healthy control (data are not included in the table).

### Cortical parcellation for network analysis

FC between a predefined set of regions of interest (ROIs) was computed using correlations between band-limited power time courses in each ROI, after applying a correction for spatial leakage confounds.^20,21^ For the purposes of the connectivity analyses, functional activity across the cortex was summarized using 39 fMRI-derived cortical ROIs. We used a data-driven, functional parcellation so that the boundaries of each region could be determined by recorded functional activity, rendering them directly relevant to our analysis. The parcellation was created from a 100-dimensional group-ICA decomposition of rs-fMRI data from the first 200 participants in the Human Connectome Project.^22^ These parcels therefore represent contiguous functionally specific local modules, which enables the analysis of a comprehensible functional network. Most ROIs have a counterpart in the opposite hemisphere, apart from those on the midline; therefore, the 39 regions provide complete coverage of cortex within a relatively low-dimensional structure. A single time series was decomposed from each region to represent the functional activity of the parcel. The first principal component of all voxels in the ROI was taken after weighting by the strength of the fMRI spatial map. The data were then orthogonalized regarding all other parcels using a symmetric multivariate approach that eliminates spurious zero-lag connectivity between neighboring parcels while maintaining a close approximation to the original time series. This novel robust approach was recently applied to successfully quantify the heritability of cortical functional architecture among related healthy participants.^23^ The time series was converted into a band-limited power time course by taking the Hilbert envelope of the “broadband” signal (3–40 Hz, therefore not restricting connectivity estimation to specific frequency bands^24^) and down-sampling with a 2-second sliding window filter.^25^ This power envelope time course was then correlated between parcels for each individual, resulting in a Fisher *z*-normalized score for each connection. These communication links between cortical regions (nodes) correspond to “edges” in a graph theory network model, and this specific approach for connectivity estimation shows greater repeatability than a wide range of other choices.^20^ Group contrasts (against age-matched healthy controls) were appraised against 5,000 permutations of the group-membership labels and corrected for multiple comparisons on a per-contrast basis (thus accounting for the number of edges contributing to each group contrast). The *p* values (*p* < 0.05) corrected using family-wise error were deemed significant. Groups were then further contrasted regarding node weight, representing the sum of all edge strengths connected to each cortical parcel in turn. The resulting (per participant) vectors of 38 node weight values were compared between groups via nonparametric permutation inference as implemented in FSL's *randomise*, with family-wise correction over the maximum *t*-stat distribution to account for the number of parcels.

### Standard protocol approvals, registrations, and patient consents

All participants provided written informed consent. The study was approved by the National Research Ethics Service South Central Oxford Research Ethics Committee B (08/H0605/85) and South Central Berkshire Committee (14/SC/0083).

## Results

**Figure 1:**
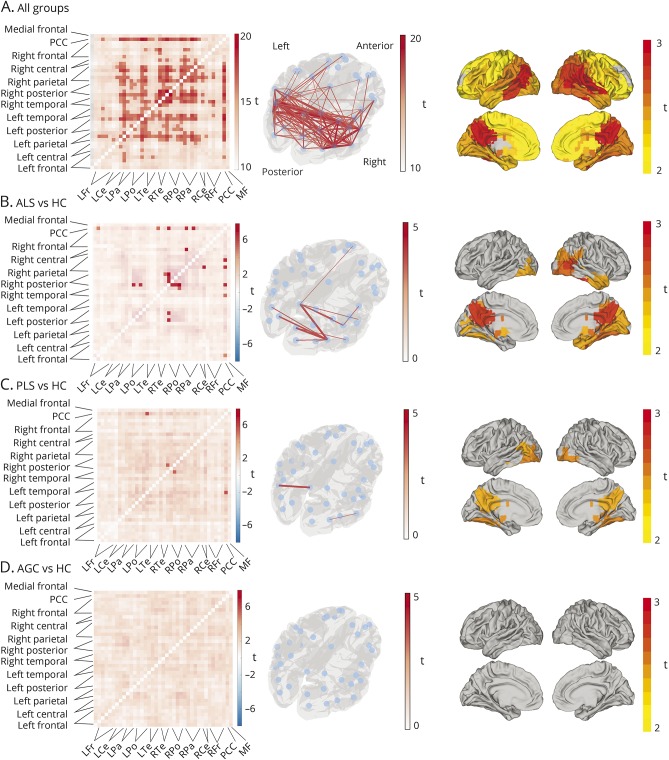
Magnetoencephalography-derived functional connectivity structure at rest and comparison between patient groups and healthy controls Matrices (left column) represent the study average (A) and group differences (B–D) in FC. Each point along the x and y axes represents a cortical parcel (or node), and each square in the matrix corresponds to an edge between nodes located on the glass brain (middle column). Values within the squares are the outputs of a parametric statistical comparison performed separately on each edge, using a generalized linear model framework to contrast the FC (calculated using correlation in band-limited power time course) across patient groups. Edges are compared against zero (A) or between groups (B–D). Group contrasts surviving multiple comparison correction are presented as unmasked squares (edges) in the matrices and lines displayed in red on the glass brains. Group comparison against HCs revealed global increases in FC in all 3 study participant groups. After multiple comparison correction, edges between the PCC and posterior nodes remained significantly stronger than healthy controls in both patients with ALS and PLS, but not AGCs (edges with no difference masked, significant edges family-wise error *p* < 0.05 unmasked, color denotes *t* value, red = stronger FC in patient groups, blue = stronger in healthy controls). Right column depicts node weight for the study average (top) and group contrasts. Group comparisons reveal significantly increased node weight in the patient groups within cortical parcels highlighted on the surface plots. AGC = asymptomatic gene mutation carrier; ALS = amyotrophic lateral sclerosis; FC = functional connectivity; HC = healthy control; PCC = posterior cingulate cortex; PLS = primary lateral sclerosis.

Relative to healthy controls, FC was increased throughout the cortical networks in patients with ALS and, to a lesser extent, in patients with PLS and AGCs. Connectivity matrices representing the study average and group differences in FC are presented in figure 1.

The FC differences were most marked in the group of patients with ALS. A degree of spatial selectivity was noted; FC increases particularly involved edges from the posterior cingulate cortex (including the connection to the right motor cortex) and edges within the right visual and occipital regions. The patients with PLS showed more subtle increases in FC, particularly between posterior and parietal nodes bilaterally. No difference in increases in FC was noted in the AGCs, affecting an overlapping spatial distribution. No edges showed decreased FC relative to age-matched healthy controls.

The group differences were further confirmed in consideration of node weight. Global connectivity to the posterior cingulate cortex and other posterior cortical parcels was significantly increased in the ALS group relative to healthy controls; similar but less extensive findings were revealed in the PLS group but not significantly so among the AGCs. Clinical measures (disease duration, rate of progression, ALS Functional Rating Scale–Revised, upper motor neuron burden, and cognition) did not correlate significantly with the connectivity strengths as described by a global mean of the interparcel edges.

## Discussion

Both ALS and PLS phenotypes showed widespread increases in FC, as measured by the degree of correlation between band-limited power envelopes across spatially distributed parcellated regions of cortex. Limited evidence was obtained in support of alterations to FC prior to the development of overt symptoms. Such abnormalities may have considerable mechanistic importance given the key role of neural oscillations in the long-range synchronization of information across the brain and might contribute to the development of accessible multimodal biomarkers, which remain a key unmet need of therapeutic trials in ALS.

Given the range of FC alterations reported in ALS via rs-fMRI,^26^ further discordance in FC degree and distribution is not unexpected with a change in neuroimaging modality, particularly considering the diverse neural activity, including modulation of oscillatory power, that may contribute to the BOLD (blood oxygen level–dependent) response. Early reports suggested a mismatch between ALS-related deficits in structural connectivity and apparently preserved functional organization,^27^ but a more complex pattern emerged with changes to FC extending beyond the primary motor cortices.^28,29^ Fewer studies have considered patients with PLS. A seed-based approach detected increased FC, notably cerebrocerebellar,^30^ while an ICA-based approach detected increased FC in the sensorimotor network (that corresponded to structural pathology) and increased FC to the right superior frontal gyrus (statistically correlating with cognitive impairment).^31^ The present findings therefore reaffirm shared pathophysiologic mechanisms between ALS and PLS.

Increased FC in ALS has been hypothesized to result from loss of intracortical inhibitory influence,^32^ supported in vivo by neurophysiology findings of accentuated cortical beta-desynchronization during movement preparation^15^ and diminished postmovement beta-rebound.^33^ Studies in healthy volunteers combining magnetic resonance spectroscopy with rs-fMRI have found evidence of the balance of glutamatergic and GABAergic neurotransmitter concentrations influencing the strength of FC.^34^ These findings may, however, also reflect direct modulation by GABA of the hemodynamic response function, rather than solely alterations to the underlying neural activity.^35^

FC changes extending beyond the primary motor networks are in keeping with ALS as a multisystem cerebral disorder with considerable cortical burden beyond the core motor phenotype.^36^ Increased FC of the precuneus and parietal cortex was previously described,^28^ and the occipital poles are reported to acquire a stronger network prominence in ALS as measured by the voxel-wise graph theory FC metric, degree centrality.^37^ FC increases related to the sensorimotor cortex may only be prominent earlier in the symptomatic course of ALS, being notably present in a study of patients without corticospinal tract diffusion tensor imaging changes.^38^ Rather than being directly pathogenic, FC increases might therefore alternatively reflect a physiologic response to functionally compensate for early pathology, which becomes overwhelmed with further disease progression. This would be in keeping with the more marked FC increases between cortical regions less vulnerable to structural damage in motor neuron disease, i.e., posterior and parietal nodes, that was demonstrated in both patients with ALS and PLS in the present study. However, no corresponding increase in sensorimotor FC is noted within the AGC group, though the sensitivity to detect such changes in a small and genetically heterogeneous group is likely to be low.

A broad (4–30 Hz) frequency band was selected for the power envelope, since most resting-state structure and connectivity is present within theta, alpha, and beta bands.^17^ Appraisal of multiple, specific frequency bands would necessitate further multiple comparisons correction and (particularly in higher gamma-frequency ranges) potentially diminish signal-to-noise ratio. The FC analysis was repeated after data decomposition into 3 frequency bands familiar to clinicians: theta (4–8 Hz), alpha (8–12 Hz), and beta (15–30 Hz). FC strength differences between patients with ALS and healthy controls were not restricted to any one frequency band, reaffirming the utility of the preceding broadband reduction (figure e-1, links.lww.com/WNL/A358). Furthermore, the selection of nodes for the parcellation analysis could alternatively be guided by anatomical landmarks, but this may have negative consequences since multiple oscillatory sources might be combined within one anatomical parcel or split across a functionally irrelevant anatomical divide. Selecting neural sources on the basis of decomposed functional data is therefore closer to a physiologic parcellation, but uncertainty remains over the total number of components to be raised—this remains a balance between interpretability, uniqueness, and potentially arbitrary subdivision of sources (of note, MaxFilter software significantly reduces the dimensionality of MEG data). Inevitably, therefore, the selection of an RSN “of interest” is nontrivial, and the interpretation of the functional significance of alterations in FC within and beyond RSNs remains speculative.

The acquisition took place as participants rested with eyes open, as fixation is desirable to minimize saccade artifacts in the MEG signal, but many rs-fMRI datasets, including our group's most recent analysis, have been conducted with eyes closed. Since Berger's (1873–1941) description, posterior alpha power is notably increased on closing the eyes; the effect on band-limited envelope coherence is not certain but likely to be positive for occipital regions at least. Whether this would differentially influence clinical groups remains to be shown. ALS, in common with most neurodegenerative diseases, causes a subtle slowing of dominant alpha, contributing to increased low-frequency power. These differences were appraised in the current resting-state MEG dataset but not found to be significant after the same family-wise error correction for number of parcels included.

A further potential confound could arise from insufficient matching of participants according to intellectual achievement,^39^ partially mitigated by reliance on spouses volunteering for inclusion as healthy controls. Finally, CNS active medications have the potential to accentuate the group difference in FC. Patients continued any regular medications for the study since withdrawal of agents providing symptomatic relief was considered unethical. Most importantly, approximately half of the patients with ALS were taking riluzole on a long-term daily basis. However, splitting the ALS data according to riluzole use did not reveal any marked differences in FC, in keeping with the short-term effect on cortical hyperexcitability as measured by transcranial magnetic stimulation.^40^

This study reinforces previous rs-fMRI findings of increased FC in ALS and builds on recent MEG data implicating cortical hyperexcitability.^15^ The directionally similar findings in carriers of high-risk genetic mutations suggests these changes may also predate the onset of symptoms. Larger cohort numbers may prove more successful in distinguishing the clinical effect of FC increases, and longitudinal repetition is a prerequisite to full realization of any prognostic capability. MEG is a well-tolerated, noninvasive, and easily repeatable investigation with potential to add sensitivity to biomarker-driven neuroprotective studies, strengthening the envisaged multimodal characterization of presymptomatic ALS. MEG therefore provides a plausible candidate metric for ALS, in the form of cortical FC, that may permit pharmacodynamic appraisal of future therapeutic interventions aimed at restoring seemingly harmful cortical hyperexcitability.

## Glossary

AGC: asymptomatic gene mutation carrier
ALS: amyotrophic lateral sclerosis
FC: functional connectivity
ICA: independent component analysis
MEG: magnetoencephalography
PLS: primary lateral sclerosis
ROI: region of interest
rs-fMRI: resting-state fMRI
RSN: resting-state network
